# NRBF2 homodimerization by its coiled-coil domain strengthens association with the PtdIns3K complex mediated by the MIT domain to promote autophagy

**DOI:** 10.1080/15548627.2025.2580438

**Published:** 2025-11-12

**Authors:** Na Li, Xiaohua Li, Xianxiu Qiu, Xuehua Pan, Shuai Wu, Jingyi Chen, Rong Liu, Jiahong Lu, Zhenyu Yue, Yanxiang Zhao

**Affiliations:** a Department of Applied Biology and Chemical Technology, The Hong Kong Polytechnic University, Hong Kong, P. R. China; b The Hong Kong Polytechnic University Shenzhen Research Institute, Shenzhen, P.R. China; c Institute of Advanced Biotechnology, Institute of Homeostatic Medicine and School of Medicine, Southern University of Science and Technology, Shenzhen, P. R. China; d Guangdong Provincial Key Laboratory of Medical Molecular Diagnostics, Dongguan Key Laboratory of Medical Bioactive Molecular Developmental and Translational Research, Guangdong Medical University, Dongguan, P.R. China; e Shenzhen Pengcheng Biopharm Co., Ltd, Shenzhen, P.R. China; f Department of Biophysics and Biophysical Chemistry, Johns Hopkins University School of Medicine, Baltimore, MD, USA; g Department of Nutrition and Health, China Agricultural University, Beijing, P.R. China; h State Key Laboratory of Quality Research in Chinese Medicine, Institute of Chinese Medical Sciences, University of Macau, Macau, P. R. China; i Department of Neurology and Neuroscience, Friedman Brain Institute, Icahn School of Medicine at Mount Sinai, New York, NY, USA

**Keywords:** Autophagy, coiled-coil, MIT, NRBF2, PIK3R4/VPS15, PtdIns3K

## Abstract

The mammalian class III phosphatidylinositol-3-kinase complex (PtdIns3K) forms two biochemically and functionally distinct subcomplexes including the ATG14-containing complex I (PtdIns3K-C1) and the UVRAG-containing complex II (PtdIns3K-C2). Both subcomplexes adopt a V-shaped architecture with a BECN1-ATG14 or UVRAG adaptor arm and a PIK3R4/VPS15-PIK3C3/VPS34 catalytic arm. NRBF2 is a pro-autophagic modulator that specifically associates with PtdIns3K-C1 to enhance its kinase activity and promotes macroautophagy/autophagy. How NRBF2 exerts such a positive effect is not fully understood. Here we report that NRBF2 binds to PIK3R4/VPS15 with moderate affinity through a conserved site on its N-terminal MIT domain. The NRBF2-PIK3R4/VPS15 interaction is incompatible with the UVRAG-containing PtdIns3K-C2 because the C2 domain of UVRAG outcompetes NRBF2 for PIK3R4/VPS15 binding. Our crystal structure of the NRBF2 coiled-coil (CC) domain reveals a symmetric homodimer with multiple hydrophobic pairings at the CC interface, which is in distinct contrast to the asymmetric dimer observed in the yeast ortholog Atg38. Mutations in the CC domain that rendered NRBF2 monomeric led to weakened binding to PIK3R4/VPS15 and only partial rescue of autophagy deficiency in *nrbf2* knockout cells. In comparison, NRBF2 with its CC domain replaced by a dimeric Gcn4 module showed proautophagic activity comparable to wild type while NRBF2 carrying a tetrameric Gcn4 module showed further enhanced activity. We propose that the oligomeric state of NRBF2 mediated by its CC domain is critical for strengthening the moderate NRBF2-PIK3R4/VPS15 interaction mediated by its MIT domain to fully activate PtdIns3K-C1 and promote autophagy.

**Abbreviations:** ATG: autophagy related; ATG14: autophagy related 14; BECN1: beclin 1; CC: coiled-coil; dCCD: delete CCD; dMIT: delete MIT; Gcn4: general control nonderepressible 4; ITC: isothermal titration calorimetry; IP: immunoprecipitation; KO: knockout; MAP1LC3/LC3: microtubule associated protein 1 light chain 3; MIM: MIT-interacting motif; MIT: microtubule interacting and trafficking; NMR: nuclear magnetic resonance; NRBF2: nuclear receptor binding factor 2; PtdIns3K: class III phosphatidylinositol 3-kinase; PtdIns3P: phosphatidylinositol-3-phosphate; PIK3C3/VPS34: phosphatidylinositol 3-kinase catalytic subunit type 3; PIK3R4/VPS15: phosphoinositide-3-kinase regulatory subunit 4; SQSTM1/p62: sequestosome 1; UVRAG: UV radiation resistance associated; VPS: vacuolar protein sorting; WT: wild type.

## Introduction

The class III phosphatidylinositol 3-kinase (PtdIns3K) complex is an evolutionarily conserved multi-protein assembly that specifically produces phosphatidylinositol-3-phosphate (PtdIns3P) by phosphorylating phosphatidylinositol at the 3’ position of the inositol ring [1–4]. The kinase activity of the PtdIns3K complex is essential for autophagy and endocytic trafficking to lysosomes and vacuoles because membrane vesicles involved in the early stages of these processes, namely autophagosomes and endosomes, are particularly enriched in PtdIns3P produced by PtdIns3K [1–4]. PtdIns3P serves as a molecular marker to recruit downstream PtdIns3P-binding effectors like PROPPINs and EEA1 to promote autophagy and endolysosomal trafficking [4,5].

In mammals, the PtdIns3K complex mainly exists in two biochemically and functionally distinct forms termed complex I (PtdIns3K-C1) and complex II (PtdIns3K-C2) [2–4,6]. Both complexes share the same three core members including the lipid kinase PIK3C3/VPS34, its endogenous binding partner PIK3R4/VPS15 and a scaffolding molecule BECN1 (beclin 1) but differ by the fourth member being either ATG14 or UVRAG. These two regulators bind to BECN1 in a mutually exclusive manner and lead to the formation of ATG14-containing PtdIns3K-C1 or UVRAG-containing PtdIns3K-C2 [3,6]. PtdIns3K-C1 is targeted mostly to nascent phagophore assembly sites at the early stage of autophagy to promote biogenesis of autophagosomes [3,6]. In comparison, PtdIns3K-C2 is mainly directed to endolysosomal compartments to facilitate maturation of autophagosomes and endosomes [3,6]. PtdIns3K-C1 and -C2 are evolutionarily conserved in yeast, with Vps30/Atg6, Atg14 and Vps38 identified as yeast orthologs for BECN1, ATG14 and UVRAG, respectively [7,8]. Crystal structure of yeast PtdIns3K-C2 and cryo-EM studies of mammalian PtdIns3K-C1 reveal that both complexes adopt the same V-shaped architecture with the PIK3C3/VPS34-PIK3R4/VPS15 subcomplex and the BECN1-ATG14 subcomplex (Vps30-Vps38 for yeast) forming the catalytic and adaptor “arm” respectively [9,10].

NRBF2 is a pro-autophagic modulator that specifically associates with PtdIns3K-C1 [11]. NRBF2 significantly enhances the lipid kinase activity of PtdIns3K-C1 and this elevated activity is critical for effective autophagy initiation [12,13]. NRBF2 can act as a RAB7 effector and regulate the interaction between PtdIns3K-C1 and CCZ1-MON1A complex, a RAB7 GEF, to promote autophagosome maturation [14]. *nrbf2* knockout mice show focal liver necrosis and suffered more severe intestinal inflammation during colitis [12,15]. NRBF2 is also linked to Alzheimer disease (AD) as it mediates autophagic degradation of amyloid beta precursor protein (APP) and its protein level is reduced in brains of 5xFAD transgenic AD mice [16,17].

NRBF2 contains an N-terminal microtubule interacting and trafficking (MIT) domain that is essential for PtdIns3K-C1 binding and a C-terminal coiled-coil (CC) domain that mediates its homodimerization [11]. Its yeast homolog Atg38 shows similar domain structures [18]. While the crystal structure of the CC domain of yeast ortholog Atg38 and cryo-EM structures of PtdIns3K-C1 in complex with NRBF2 have been reported, molecular details of how NRBF2 specifically associates with PtdIns3K-C1 to promote autophagy are still not fully understood [11,19,20].

Here we report that NRBF2 does not have direct interactions with BECN1 or ATG14, two members of PtdIns3K-C1. Instead, it binds to PIK3R4/VPS15 with moderate affinity through a conserved site on its MIT domain. NRBF2 is compatible with PtdIns3K-C1 but not the UVRAG-containing PtdIns3K-C2 because NRBF2 and UVRAG bind to overlapping sites on PIK3R4/VPS15. Our crystal structure of the NRBF2 CC domain reveals a symmetric homodimer that is in distinct contrast to the asymmetric dimer observed in yeast ortholog Atg38. The oligomeric state of NRBF2 mediated by its CC domain is important for binding to PIK3R4/VPS15 because monomeric NRBF2 showed weakened binding to PIK3R4/VPS15 and partial rescue of the autophagy deficiency in *nrbf2* knockout cells while engineered dimeric or tetrameric NRBF2 constructs showed stronger binding to PIK3R4/VPS15 and elevated autophagic activity. We propose that the oligomeric state of NRBF2 mediated by its CC domain is critical for strengthening the NRBF2-PIK3R4/VPS15 interaction mediated by its MIT domain in order to fully activate PtdIns3K-C1 and promote autophagy.

## Results

### NRBF2 interacts with the PIK3C3/VPS34-PIK3R4/VPS15 subcomplex but not BECN1 or ATG14 within PtdIns3K-C1

Previous studies using hydrogen-deuterium exchange mass spectroscopy (HDX-MS) suggested that NRBF2 could contact multiple sites within PtdIns3K-C1 including the N-terminal domains of BECN1 and PIK3C3/VPS34, the CC domain of ATG14 and the helical solenoid region of PIK3R4/VPS15 because NRBF2 affected the HDX profile of these regions [11,19]. Co-immunoprecipitation (co-IP) studies also showed that NRBF2 could be pulled down by any member of PtdIns3K-C1 including BECN1, ATG14 and PIK3C3/VPS34 [12,13]. However, methods like HDX-MS and co-IP are not specific for detecting direct interactions because HDX profiles can be influenced by conformational changes while all members of a stable complex can be immunoprecipitated together even though they do not interact directly. Thus, biochemical assays that use purified protein samples to measure direct interactions are needed to confirm which members of PtdIns3K-C1 are direct binding partners for NRBF2.

**Figure 1.**
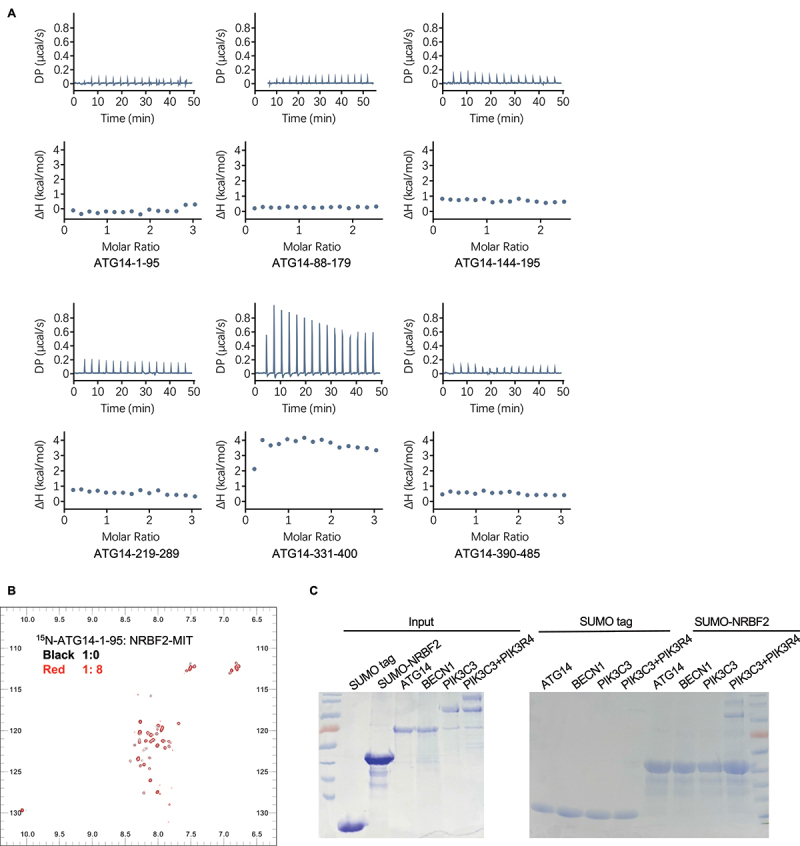
NRBF2 does not have direct interactions with BECN1 or ATG14. (A) ITC profiles to assess the direct interaction between NRBF2 and ATG14. No bindings were detectable. (B) ^1^H-^15^N HSQC NMR spectra of ^15^N-labeled ATG14-1–95 in the absence (black) or presence of NRBF2 MIT domain at 8x molar excess (red). No chemical shifts were observed. (C) Affinity-isolation assay to assess the direct binding between NRBF2 and purified full-length BECN1, ATG14, PIK3C3/VPS34 and the PIK3C3/VPS34-PIK3R4/VPS15 complex. SUMO-tagged NRBF2 only pulled down the PIK3C3/VPS34-PIK3R4/VPS15 complex but not BECN1, ATG14 or PIK3C3/VPS34.

We carried out isothermal titration calorimetry (ITC) experiments to assess whether NRBF2 interacts directly with ATG14, the unique member of PtdIns3K-C1, and BECN1, another essential scaffolding member. We used a truncated version of NRBF2 with its C-terminal flexible loop removed (NRBF2-Δ-211–287) to represent full-length NRBF2 because the loop region is functionally dispensable and readily degrades during the purification process. As full-length ATG14 was not soluble when expressed alone, we generated six soluble constructs to represent the N-terminal region (residues 1–95), two CC fragments (residues 88–179 and 144–195), the BARA-like domain (two constructs covering residues 219–289 and 331–400 respectively) and the C-terminal BARKOR/ATG14 autophagosome targeting sequence (BATS; residues 390–485). Several studies have reported that upon starvation signal, the N-terminal region of ATG14 would become phosphorylated to promote autophagy [21,22]. Thus, four mutant versions of the ATG14 N-terminal region (residues 1–95) with single (S61E, S83E), double (S61E S83E) and triple (T48E T49E T54E) mutations were generated to mimic such phosphorylation. We also generated three soluble BECN1 constructs to represent the N-terminal BH3-containing region (residues 1–105), the central CC domain (residues 174–266), and the C-terminal BARA domain (residues 243–448) (Figure S1). However, none of these ATG14 or BECN1 constructs showed any detectable binding to NRBF2 (Figure 1A and Figure S1).

We also carried out NMR titration studies to characterize the interaction between the MIT domain of NRBF2 and the N-terminal region of ATG14 (residues 1–95). The ^1^H-^15^N HSQC spectrum of ^15^N-labeled ATG14-1–95 showed poor dispersion, in agreement with the predicted flexible conformation for this region (Figure 1B). Titrating increasing amount of NRBF2-MIT into ATG14-1–95 did not induce any observable chemical shifts, even when the NRBF2:ATG14 molar ratio reached 8:1 (Figure 1B).

As these ITC and NMR titration studies used only fragments of BECN1 and ATG14, we decided to carry out further *in vitro* affinity-isolation studies using full-length constructs. Similar to previous studies, we transiently expressed and purified full-length BECN1, ATG14, PIK3C3/VPS34 and the PIK3C3/VPS34-PIK3R4/VPS15 complex from suspension Expi293F cells (Figure 1C). Full-length PIK3R4/VPS15 could not be purified alone and must be co-expressed with PIK3C3/VPS34 to form the PIK3C3/VPS34-PIK3R4/VPS15 complex. Our data shows that SUMO-tagged NRBF2 only pulled down the PIK3C3/VPS34-PIK3R4/VPS15 complex but not BECN1, ATG14 or PIK3C3/VPS34 (Figure 1C). This data suggests that PIK3R4/VPS15 is the key member within PtdIns3K-C1 that directly interacts with NRBF2. Notably, our data differs from a previous study by Lu *et. al*., which showed that GST-tagged NRBF2 pulled down a small amount of FLAG-tagged ATG14 as detected by western blotting [12]. Our study is more robust on two aspects. First, we used Coomassie Brilliant Blue to detect the amount of protein pulled down by NRBF2, which is more stringent than the western blotting method used by Lu *et. al*. in terms of filtering out nonspecific or weak interactions. Secondly, our data compared the pull-down result for BECN1, ATG14, PIK3C3/VPS34 and the PIK3C3/VPS34-PIK3R4/VPS15 complex while Lu *et. al*. only assessed ATG14. Overall, our *in vitro* ITC binding assays, NMR titration studies and affinity-isolation results confirm that NRBF2 interacts with the PIK3C3/VPS34-PIK3R4/VPS15 subcomplex, i.e., the catalytic arm of the V-shaped PtdIns3K-C1 but not the BECN1-ATG14 scaffolding arm. Specifically, NRBF2 may only bind to PIK3R4/VPS15 within the PIK3C3/VPS34-PIK3R4/VPS15 subcomplex as it does not pull down PIK3C3/VPS34 alone. Overall, our *in vitro* binding studies confirm that NRBF2 only has direct interaction with PIK3R4/VPS15 within PtdIns3K-C1 but not other members such as BECN1, ATG14 or PIK3C3/VPS34.

### NRBF2 binds to PIK3R4/VPS15 through a conserved MIM2 site located between the first and the third helix of its MIT domain

The N-terminal MIT domain of NRBF2 is essential for PtdIns3K-C1 binding as NRBF2 constructs with this domain deleted failed to pull down members of PtdIns3K-C1 in co-IP experiments [12,13]. In the cryo-EM structure of PtdIns3K-C1 in complex with NRBF2, one MIT domain of the NRBF2 homodimer was seen docked onto the helical solenoid domain of PIK3R4/VPS15 near the base of the V-shaped PtdIns3K-C1 [20]. However, atomic details of this NRBF2-PIK3R4/VPS15 interaction are not clear due to limited resolution of ~7–8 Å [20].

**Figure 2.**
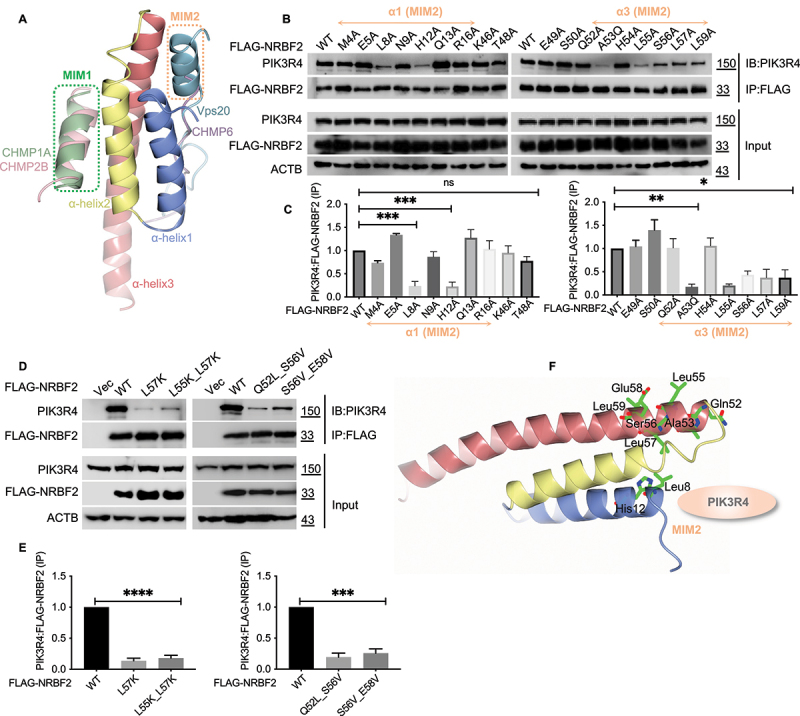
NRBF2 binds to PIK3R4/VPS15 through a conserved site located between the first and third helix of its MIT domain. (A) The MIT-interacting motifs of CHMP1A (green, PDB id 2JQ9), CHMP2B (pink, PDB id 2JQK), CHMP6 (purple, PDB id 2K3W), and Vps20 (light blue, PDB id 5FVL) are mapped onto the NRBF2 MIT domain after structural superposition of their respective MIT domains. α1 to α3: helix 1 to 3 of the NRBF2 MIT domain. The two distinct binding sites are marked MIM1 and MIM2. (B) Co-IP experiments to map the PIK3R4/VPS15 binding site on the NRBF2 MIT domain. FLAG-tagged NRBF2 mutants were transfected into HEK293T cells and their interaction with endogenous PIK3R4/VPS15 was probed using anti-FLAG M2 magnetic beads for immunoprecipitation (IP), followed by immunoblotting (IB) using anti-PIK3R4/VPS15 antibody. Mutations in α1 and α3 of the NRBF2 MIT domain weakened the NRBF2-PIK3R4/VPS15 interaction while mutations in α2 had little effect. (C) Quantification of PIK3R4/VPS15:FLAG-NRBF2 protein levels from data in (B). (D) Similar co-IP experiments as (B), with single and double mutations in α3 to completely abolish the NRBF2-PIK3R4/VPS15 interaction. (E) Quantification of PIK3R4/VPS15:FLAG-NRBF2 protein levels from data in (D). (F) A structural model mapping the PIK3R4/VPS15 binding site on the NRBF2 MIT domain. The NRBF2 MIT domain is depicted as docking onto the helical domain of PIK3R4/VPS15, with residues identified by co-IP data in (B) and (D) as critical for PIK3R4/VPS15 binding highlighted in ball-and-stick model.

The MIT domain is a conserved structural module frequently found in proteins involved in vacuolar sorting and endocytic trafficking like PIK3C3/VPS34, SPART (spartin) and VTA1/LIP5 [23–25]. Like other MIT domains, the NRBF2 MIT domain consists of three α-helices (helix α1 to α3) that fold into a three-helix bundle (Figure 2A) [23–25]. MIT domains can bind a variety of sequence motifs through two distinct binding sites on their surface (Figure 2A) [23]. One site is formed between α2 and α3 while the cognate peptide is usually a short α-helix with the MIT-interacting Motif 1 (MIM1) of (D/E)xxLxxRLxxL(K/R) [23]. The other site is formed between α1 and α3 and the bound peptides often adopt extended conformation with the MIT-interacting Motif 2 (MIM2) of (L/V)Px(V/L)P [23].

We decided to conduct mutational scanning to identify key residues in the NRBF2 MIT domain for PIK3R4/VPS15 binding. Firstly, we did alanine scanning for most residues involved in the MIM1 and MIM2 sites while residue A53 was mutated to Q. Our co-IP results show that mutating residues on α1 (L8A and H12A) and α3 (A53Q, L55A, S56A, L57A, and L59A) within the MIM2 site significantly weakened or abolished PIK3R4/VPS15 binding (Figure 2B,C). In contrast, mutations around the MIM1 site (T48A, E49A and S50A on α2 and H54A on α3) had little impact (Figure 2B). We then focused on the key residues on MIM2 site and did further mutations such as L-to-K for L55 and L57 to abolish the hydrophobic interactions and Q/S/E-to-L/V for Q52, S56 and E58 to perturb possible polar interactions. All these mutants showed significantly reduced binding to PIK3R4/VPS15 (Figure 2D,E).

Overall, the PIK3R4/VPS15 binding site on the NRBF2 MIT domain is the MIM2 type and covers a small region that only involves the first two helical turns of α1 and α3 (Figure 2F). This same region was seen docked onto the helical domain of PIK3R4/VPS15 in the low-resolution cryo-EM structure of the NRBF2-containing PtdIns3K-C1 complex [20]. Notably, the MIM2 binding site on MIT domains usually involves a long surface groove between α1 and α3 to enhance specificity and potency [23]. For example, the MIM2 site on the MIT domain of VPS4 involves a large surface groove and multiple interactions to achieve potent and specific binding to CHMP6 [26]. The small binding region we identified may explain the moderate binding affinity for the NRBF2-PIK3R4/VPS15 interaction. As a result, NRBF2 serves as only an accessory modulator of PtdIns3K-C1 but not a stable core member like ATG14 or BECN1.

### The NRBF2-PIK3R4/VPS15 interaction is incompatible with PtdIns3K-C2 due to competition with C2 domain of UVRAG

When the cryo-EM structure of PtdIns3K-C1 in complex with NRBF2 was superimposed with the crystal structure of the yeast PtdIns3K-C2, the MIT domain of NRBF2 and the C2 domain of Vps38 appeared to dock onto the same region on PIK3R4/VPS15 [9,20]. However, given the low resolution of both structures, we decided to validate whether UVRAG competes against NRBF2 for binding to endogenous PIK3R4/VPS15.

In our previous studies, we used co-IP experiments to investigate the competition between ATG14 and UVRAG for binding to endogenous BECN1 [27]. Using similar approach, we decided to check whether NRBF2 and UVRAG would compete for binding to endogenous PIK3R4/VPS15 to form the NRBF2-containing PtdIns3K-C1 and UVRAG-containing PtdIns3K-C2 respectively. In our competitive co-IP experiments, the “bait” protein is FLAG-tagged while the competitor is GFP-tagged. Both the “bait” and the “competitor” are transiently over-expressed in HEK293T cells and the amount of endogenous PIK3R4/VPS15 and PIK3C3/VPS34 pulled down by the “bait” is assessed by standard co-IP.

**Figure 3.**
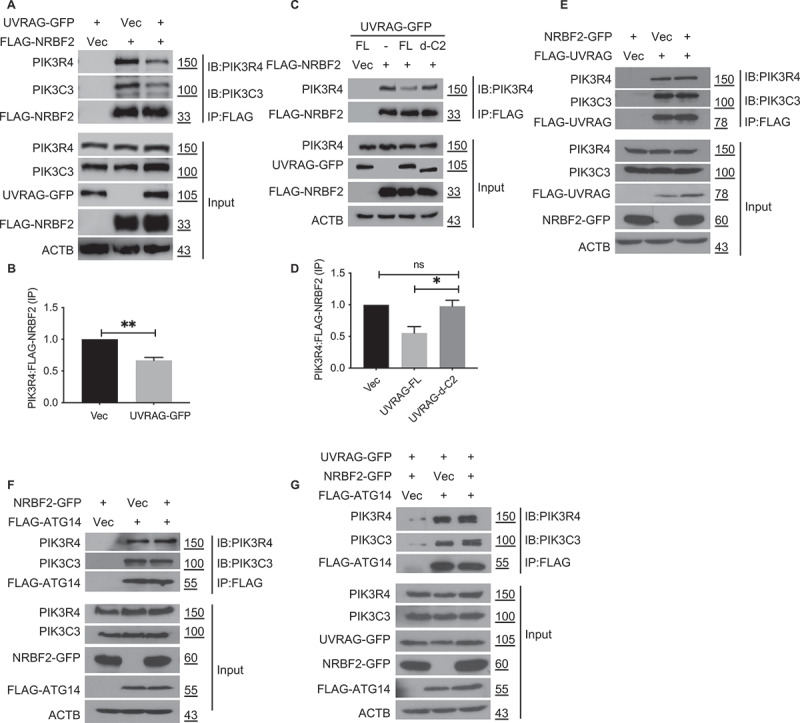
NRBF2 and UVRAG compete for PIK3R4/VPS15 binding through the C2 domain of UVRAG. (A) Co-IP experiments to assess the competition between NRBF2 and UVRAG for PIK3R4/VPS15 binding. FLAG-NRBF2 and UVRAG-GFP were co-transfected into HEK293T cells. The association of NRBF2 with endogenous PIK3C3/VPS34 and PIK3R4/VPS15 was probed using anti-FLAG M2 magnetic beads for immunoprecipitation (IP), followed by immunoblotting (IB) using anti-PIK3C3/VPS34 and anti-PIK3R4/VPS15 antibody. (B) Quantification of PIK3R4/VPS15:FLAG-NRBF2 protein levels from data in (A). (C) Similar co-IP experiments as (A), but different UVRAG constructs were used. FL: full-length. d-C2: C2 domain deleted. (D) Quantification of PIK3R4/VPS15:FLAG-NRBF2 protein levels from data in (C). (E) Similar Co-IP experiments as (A), but the tags on NRBF2 and UVRAG were swapped. (F) Co-IP experiments to assess the impact of NRBF2 on the interaction of ATG14 with PIK3C3/VPS34 and PIK3R4/VPS15. NRBF2-GFP was co-transfected with FLAG-ATG14 into HEK293T cells. The interaction between FLAG-ATG14 and endogenous PIK3C3/VPS34 or PIK3R4/VPS15 was probed using anti-FLAG M2 magnetic beads for immunoprecipitation (IP), followed by immunoblotting (IB) using anti-PIK3C3/VPS34 or anti-PIK3R4/VPS15 antibody. (G) Similar co-IP experiment as (F), with UVRAG-GFP added in co-transfection to assess whether NRBF2 impacts the competition between ATG14 and UVRAG.

Our data shows that the GFP-tagged UVRAG as “competitor” reduced the amount of endogenous PIK3R4/VPS15 pulled down by FLAG-tagged NRBF2 (Figure 3A,B). This data confirms that UVRAG is a stronger binding partner for PIK3R4/VPS15 than NRBF2, possibly because UVRAG interacts with both BECN1 and PIK3R4/VPS15 within PtdIns3K-C2 while NRBF2 only interacts with PIK3R4/VPS15 in PtdIns3K-C1. Our data also shows that the GFP-tagged UVRAG with C2 domain deleted as “competitor” did not affect the amount of endogenous PIK3R4/VPS15 pulled down by NRBF2 (Figure 3C,D). This data confirms that the C2 domain of UVRAG directly competes against NRBF2 for PIK3R4/VPS15 binding, likely because they bind to the same site on PIK3R4/VPS15. Furthermore, the GFP-tagged NRBF2 as “competitor” did not affect the amount of endogenous PIK3R4/VPS15 pulled down by UVRAG (Figure 3E). This data suggests that NRBF2 is a weaker binding partner for PIK3R4/VPS15, which further corroborates our co-IP data showing that UVRAG is a stronger binding partner than NRBF2 (Figure 3A,B). Our previous study reported that, upon over-expression, ATG14 and UVRAG competed for binding to endogenous BECN1 via their respective CC domains [27]. Such competition likely affects the relative abundance of PtdIns3K-C1 versus PtdIns3K-C2. We decided to check whether NRBF2 would help ATG14 gain competitive advantage over UVRAG and increase the abundance of PtdIns3K-C1. Our co-IP results showed that, in presence of endogenous UVRAG as a competitor, FLAG-tagged ATG14 pulled down a similar amount of endogenous PIK3R4/VPS15 and PIK3C3/VPS34 whether in presence or absence of NRBF2 over-expression (Figure 3F). We repeated this co-IP experiment using over-expressing GFP-tagged UVRAG as the competitor and got the same result (Figure 3G). These results further confirm that NRBF2 does not affect the BECN1-ATG14 subcomplex, i.e. the scaffolding arm of V-shaped PtdIns3K-C1.

### NRBF2 coiled-coil domain forms a stable and symmetric homodimer

The crystal structure of the C-terminal homodimerization domain of yeast Atg38 reveals a mushroom-like asymmetric dimer with a 4-helix cap at the N-terminal end and a long parallel CC stalk at the C-terminal [11]. The asymmetry arises from the different conformations adopted by the second helix in each monomer, being straight in one monomer and bent in the other [11]. NRBF2 and Atg38 have low sequence conservation in the coiled-coil domain [12,13,18]. It is not clear whether the asymmetric dimer structure observed in Atg38 would apply to NRBF2.

We purified the coiled-coil domain of mouse NRBF2 (aa 165–210) and confirmed that it formed a highly stable homodimer by dynamic light scattering and circular dichroism measurements (Figure S2). This construct yielded crystals that diffracted to 2.2 Å. The structure was determined by Single Isomorphous Replacement Anomalous Scattering (SIRAS) method using selenomethionine-derivatized crystals for phasing. There are 8 copies of the CC domain helix in the asymmetric unit (Figure S3). The homodimer interface was identified based on the canonical hydrophobic pairings.

**Figure 4.**
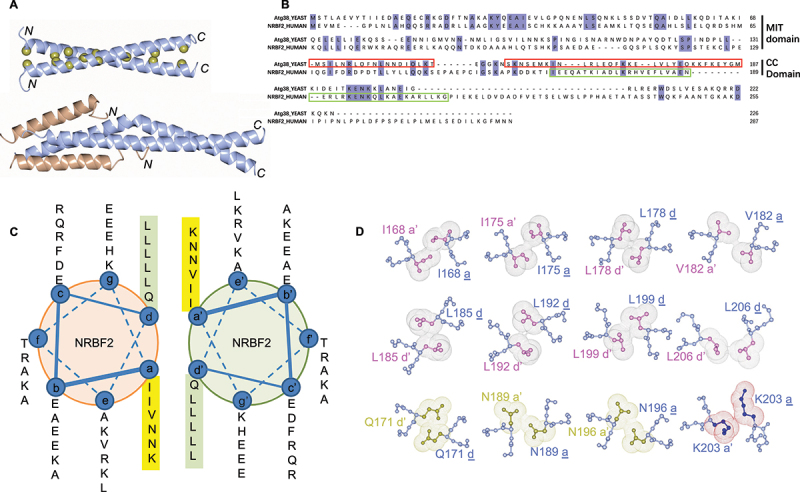
The NRBF2 CC domain forms a parallel coiled-coil homodimer with hydrophobic pairings. (A) The crystal structure of the NRBF2 CC domain reveals a parallel coiled-coil homodimer. Leucine residues on the dimer interface are highlighted as golden spheres. While the crystal structure of the C-terminal homodimerization domain of yeast Atg38 reveals a mushroom-like asymmetric dimer with a 4-helix cap at the N-terminal end and a long parallel CC stalk at the C-terminal (PDB code:5KC1). (B) Comparison of the sequences of NRBF2 and its yeast ortholog Atg38. Compared to Atg38, the NRBF2 CC domain is shorter in sequence and contains no corresponding region for the short N-terminal helix seen in Atg38. (C) Helical wheel presentation of the NRBF2 CC domain. Residues at *a* and *d* positions of the heptad repeat motifs are highlighted. (D) Close-up view of the *a-a’* and *d-d’* pairings at the dimer interface of the CC domain. Eight pairings are hydrophobic (pink) while four pairings contain polar (golden) or charged (dark blue) residues. The *a-a’* and *d-d’* pairings are illustrated by van der waals spheres depicting the side chains.

The structure of the NRBF2 CC domain reveals a homodimer with two α helices wrapped around each other in parallel fashion (Figure 4A and Figure S3). Compared to Atg38, the NRBF2 CC domain is shorter in sequence and contains no corresponding region for the short N-terminal helix seen in Atg38 (Figure 4B). The single α helix within the NRBF2 CC domain contains 6 heptad repeats of the *abcdefg* sequence motif and forms 12 *a-a’* and *d-d’* pairings at the dimer interface (Figure 4C). Eight pairings are the canonical hydrophobic type while the remaining four are the non-canonical type involving polar or charged residues (Figure 4D).

### The oligomeric state of NRBF2 is essential for strengthening its binding to PIK3R4/VPS15

While structural studies by us and others confirm that the CC domain of NRBF2 and its yeast ortholog Atg38 forms a homodimer, its functional significance is not clear. In the cryo-EM structure of PtdIns3K-C1 in complex with NRBF2, the entire CC domain was absent, thus unlikely to have any stable interaction with PtdIns3K-C1 [20]. However, several studies also showed that the NRBF2 MIT domain alone without the CC domain did not activate the lipid kinase activity of PtdIns3K-C1 as much as the NRBF2 full-length homodimer [20]. Thus, the CC domain does exert positive effect on NRBF2 function.

**Figure 5.**
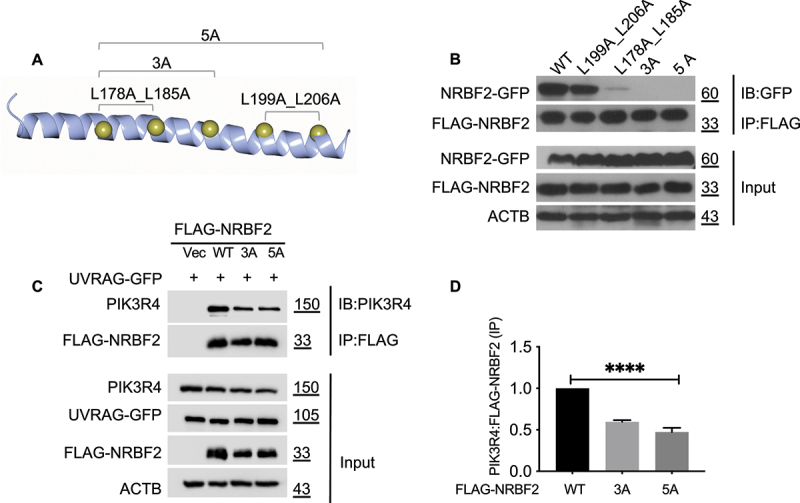
Mutations in the NRBF2 CC domain to render it monomeric lead to weakened binding to PIK3R4/VPS15. (A) Leu-to-Ala mutations in the NRBF2 CC domain targeting the five leucine zipper pairs. Double (L178A_L185A, L201A_L206A), triple (3A) and pentameric (5A) mutants were generated. (B) Co-IP experiments to assess the self-association of NRBF2 mutants. FLAG- and GFP-tagged NRBF2 constructs were co-transfected into HEK293T cells. Their self-association was probed using anti-FLAG M2 magnetic beads for immunoprecipitation (IP), followed by immunoblotting (IB) using anti-GFP antibody. Double mutant (L178A_L185A), 3A and 5A all lost self-association. (C) Co-IP experiments to assess the binding of NRBF2 mutants to endogenous PIK3R4/VPS15. FLAG-tagged NRBF2 constructs were transfected into HEK293T cells. Their binding to endogenous PIK3R4/VPS15 was probed using anti-FLAG M2 magnetic beads for immunoprecipitation (IP), followed by immunoblotting (IB) using anti-PIK3R4/VPS15 antibody. 5A pulled down significantly less of endogenous PIK3R4/VPS15 compared to the wild type. (D) Quantification of PIK3R4/VPS15:FLAG-NRBF2 protein levels from data in (C).

Guided by our structure of the NRBF2 CC domain, we decided to investigate whether the oligomeric state of NRBF2 affects its interaction with PIK3R4/VPS15. We generated several monomeric mutants of NRBF2 with 2, 3 or 5 leucine residues at the dimer interface replaced by alanine (Figure 5A). Our co-IP results reveal that mutating two leucine residues at the N-terminal region of the CC domain (L178A_L185A) significantly reduced NRBF2 self-association while mutations aimed at two leucine residues at the C-terminal region (L199A_L206A) had little effect (Figure 5B). Mutating 3 or 5 leucine residues (NRBF2_3A and _5A) completely abolished the self-association (Figure 5B). We also carried out co-IP studies to assess the impact of these mutations on the NRBF2-PIK3R4/VPS15 interaction. In presence of over-expressed UVRAG as the competitor, both NRBF2_3A and NRBF2_5A pulled down ~50% less endogenous PIK3R4/VPS15 than wild type (Figure 5C,D). This result confirms that the oligomeric state of NRBF2 as mediated by its CC domain is important to ensure full binding to PIK3R4/VPS15.

**Figure 6.**
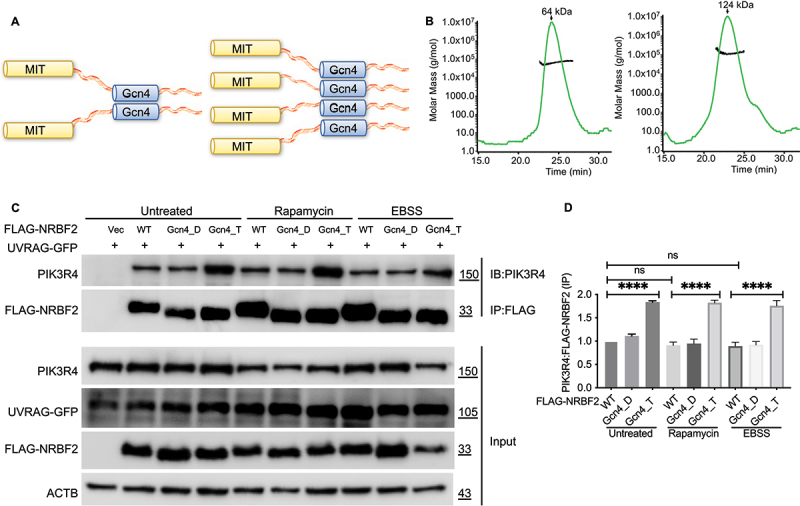
NRBF2 with its CC domain replaced with dimeric or tetrameric Gcn4 module shows strengthened binding to PIK3R4/VPS15. (A) Design scheme to generate NRBF2 constructs with its CC domain replaced by dimeric or tetrameric Gcn4 modules. (B) The oligomeric state of NRBF2_Gcn4_Dimer and _Tetramer is confirmed by dynamic light scattering profiles. The estimated molecular weight matches the expected dimeric or tetrameric state. (C) Co-IP experiments to assess the binding of NRBF2_Gcn4_Dimer and _Tetramer to endogenous PIK3R4/VPS15. FLAG-NRBF2 constructs and UVRAG-GFP were transfected into HEK293T cells. HEK293T cells were untreated or treated with 100 nM rapamycin (2 h) or treated with EBSS medium (2 h) before harvesting. Their binding to endogenous PIK3R4/VPS15 was probed using anti-FLAG M2 magnetic beads for immunoprecipitation (IP), followed by immunoblotting (IB) using anti-PIK3R4/VPS15 antibody. (D) Quantification of PIK3R4/VPS15:FLAG-NRBF2 protein levels from data in (C).

To further evaluate the functional importance of the oligomeric state of NRBF2, we replaced its CC domain with dimeric and tetrameric Gcn4 leucine zipper domains to generate NRBF2_Gcn4_Dimer and NRBF2_Gcn4_Tetramer constructs (Figure 6A). Gcn4 is a transcriptional activator that forms a homodimer through its leucine zipper domain. This domain contains 5 heptad repeats and forms a parallel CC homodimer with multiple leucine zipper pairs at the dimer interface [28]. This Gcn4 domain can be engineered to form tetrameric CC assembly by mutating all residues at *a* and *d* positions of heptad repeats to Leu and Ile (Figure S4) [29]. Previous studies have shown that the Gcn4 module can be used as a fusion partner to induce desired oligomeric state in the target protein of interest [30–32].

The oligomeric states of our NRBF2_Gcn4_Dimer and _Tetramer constructs were confirmed by dynamic light scattering experiments (Figure 6B). We then carried out co-IP experiments to assess whether these two engineered constructs retained binding to PIK3R4/VPS15. Our data shows that, under normal fed condition in presence of over-expressed UVRAG as competitor, NRBF2_Gcn4_Dimer pulled down a similar amount of endogenous PIK3R4/VPS15 as compared to wild-type NRBF2 while NRBF2_Gcn4_Tetramer pulled down noticeably more (Figure 6C,D). Similar pattern was observed after rapamycin treatment or starvation to induce autophagy (Figure 6C,D). These results suggest that the oligomeric state of NRBF2 is essential for enhancing its binding to PIK3R4/VPS15 under both normal and autophagy-inducing conditions, with the tetramer construct showing stronger interaction than the dimer. Additionally, our data further validates our previous finding that the CC domain of NRBF2 is not directly involved in binding to PIK3R4/VPS15 because it can be replaced by a different CC domain with the same or higher-order oligomeric state.

### The oligomeric state of NRBF2 is critical for promoting autophagy

**Figure 7.**
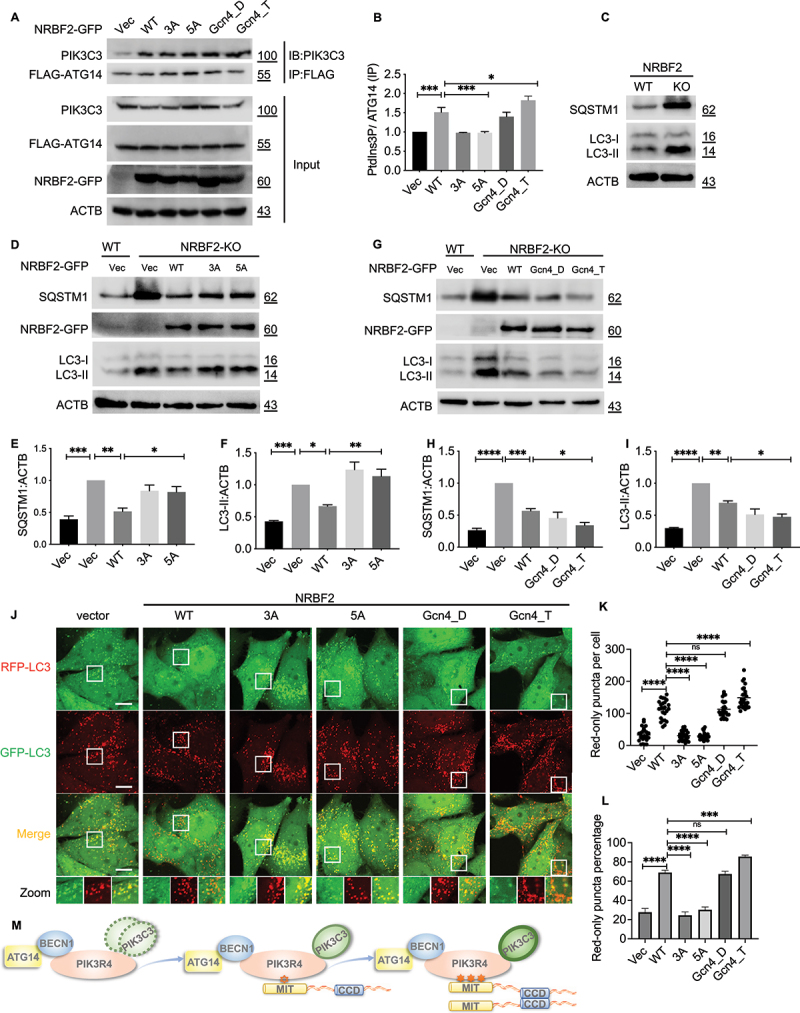
Oligomeric state of NRBF2 impacts autophagic activity. (A) N2a *nrbf2* knockout cells were transfected with NRBF2-GFP constructs and FLAG-ATG14. Atg14L-linked PIK3C3/VPS34 is pulled down by an anti-Flag antibody. (B) The quantification result shows ATG14-linked PIK3C3/VPS34 lipid kinase activity of IP products analyzed by PtdIns3P ELISA kit. (C) N2a cell line with *nrbf2* knockout shows significantly elevated level of SQSTM1 and LC3. (D) Western blots to assess the SQSTM1 and LC3 level in N2a *nrbf2* knockout cells after transfection of monomeric NRBF2 constructs including 3A and 5A. (E-F) Quantification of SQSTM1 and LC3 levels from data in (D). (G) Similar western blots as (D), but transfected with NRBF2 constructs containing the Gcn4 domain in either dimeric (Gcn4_D) and tetrameric (Gcn4_T) form. (H-I) Quantification of SQSTM1 and LC3 levels from data in (G). (J) Representative confocal fluorescence images of HeLa cells stably expressing RFP-GFP-LC3 after transient transfection with different FLAG-NRBF2 constructs. The red only puncta showed significant increase with NRBF2-WT, NRBF2_Gcn4_Dimer and _Tetramer, but not with monomeric NRBF2 constructs (3A and 5A). Scale bar: 10 μm. (K-L) Quantification results show the numbers of red-only puncta and percentage of red-only puncta from data in (J). *n* = 25 cells from 3 independent experiments. (M) Diagram to summarize the functional role of NRBF2 coiled-coil domain in strengthening association with the PtdIns3K complex mediated by the MIT domain to promote autophagy.

To investigate the functional significance of the homodimeric state of NRBF2 in autophagy, we made use of the neuroblastoma N2a cell line with *nrbf2* knockout (KO) established in our previous studies [14,16]. We first tested whether the oligomeric state of NRBF2 would affect the lipid kinase activity of PtdIns3K-C1. NRBF2 constructs with different oligomeric states were transiently transfected into N2a KO cells together with FLAG-tagged ATG14. The ATG14-containing PtdIns3K-C1 complex was pulled down by IP and its lipid kinase activity was assessed using ELISA assay to detect PtdIns3P (Figure 7A). Our data shows that dimeric constructs including wild-type and NRBF2_Gcn4_Dimer led to significantly elevated level of PtdIns3P while NRBF2_Gcn4_Tetramer showed even more prominent effect. In comparison, monomeric constructs including NRBF2_3A and _5A showed no such effect (Figure 7B).

We then set out to assess whether the oligomeric state of NRBF2 would affect the levels of autophagy markers SQSTM1/p62 and LC3-II. The N2a cell line with *nrbf2* KO is known to be deficient at the late auto-lysosomal stage, which leads to significant increase in the basal level of SQSTM1 and LC3-II (Figure 7C). Transient overexpression of NRBF2 wild-type in N2a KO cells reduced the level of endogenous SQSTM1 and LC3-II to that comparable to regular N2a cells, thus restoring autophagy activity. However, no such effect was observed for monomeric NRBF2_3A and _5A (Figure 7D-F). Furthermore, NRBF2_Gcn4_Dimer and _Tetramer also reduced the level of SQSTM1 and LC3-II, thus restoring autophagy activity similar to NRBF2 wild-type (Figure 7G-I). Notably, NRBF2_Gcn4_Tetramer showed slightly stronger effect, leading to lower level of SQSTM1 and LC3-II compared to NRBF2 wild-type (Figure 7G-I).

We also checked the cytosolic distribution of NRBF2 constructs in HeLa cells stably expressing GFP-LC3. While wild-type NRBF2 showed a diffusive pattern with a few puncta co-localized with GFP-LC3, the monomeric constructs 3A and 5A only showed diffusive pattern with no puncta observed (Figure S5). In comparison, both NRBF2_Gcn4_Dimer and NRBF2_Gcn4_Tetramer formed similar puncta as NRBF2 wild-type and showed excellent co-localization with GFP-LC3 puncta (Figure S5). We also assessed the impact of NRBF2 oligomeric state on autophagic flux using HeLa cells stably expressing RFP-GFP-LC3. Under regular condition and in absence of autophagy-inducing signals, the number of RFP-only puncta in HeLa cells is low and suggests a low basal level of autophagy (Figure 7J-L). Transient over-expression of NRBF2 wild-type as well as NRBF2_Gcn4_Dimer and _Tetramer constructs led to significant increase of RFP-only puncta, thus indicating enhanced autophagic flux. However, monomeric NRBF2_3A and _5A did not show such effect (Figure 7J-L). Overall, our results suggest that the oligomeric state of NRBF2 is critical for its cytosolic co-localization with autophagosome markers and for promoting autophagy.

## Discussion

NRBF2 is an accessory modulator of PtdIns3K-C1 and positively regulates the lipid kinase activity of this complex to promote autophagy [33]. Despite extensive studies, some critical aspects of how NRBF2 selectively associates with PtdIns3K-C1 to promote autophagy are not fully understood. Here our biochemical and structural studies offer new insight on how the MIT domain and CC domain of NRBF2 play distinct roles in mediating selective binding to PtdIns3K-C1 to promote autophagy.

Our study clarifies that NRBF2 directly binds to PIK3R4/VPS15 through its MIT domain but does not interact with BECN1 or ATG14. Previous studies using HDX-MS profiling and co-IP studies have implicated BECN1, ATG14 and PIK3C3/VPS34 as potential binding partners for NRBF2 but these experimental methods cannot differentiate between direct and indirect interactions [33]. Our *in vitro* studies including affinity-isolation assay using full-length BECN1 or ATG14 as well as ITC and NMR measurements using fragments of these two proteins clearly rule out them as possible direct binding partners for NRBF2. Instead, our *in vitro* affinity-isolation study showed that NRBF2 binds directly to PIK3R4/VPS15. Additionally, our co-IP data coupled with mutational scanning confirms that a conserved MIM2 site on the NRBF2 MIT domain is responsible for PIK3R4/VPS15 binding. Notably, this binding site is significantly smaller than the MIM2 sites identified in other MIT domains and likely renders the NRBF2-PIK3R4/VPS15 interaction of moderate affinity. Furthermore, our data shows that NRBF2 competes against the C2 domain of UVRAG for PIK3R4/VPS15 binding, thus explaining why NRBF2 is selective for PtdIns3K-C1 but incompatible with the UVRAG-containing PtdIns3K-C2 [12,13].

Our data also confirms that the oligomeric state of NRBF2 mediated by its CC domain is critical for strengthening its association with PtdIns3K-C1. Two models have been proposed to explain how the oligomeric NRBF2 binds to PtdIns3K-C1. Young *et. al*. stated that full-length NRBF2, through its coiled-coil domain, could dimerize PtdIns3K-C1 to form a dimer of pentamers [19]. This finding was based on their size-exclusion chromatography (SEC) study, which showed that the estimated molecular weight of PtdIns3K-C1 in the presence or absence of NRBF2 was ~669 kDa and ~440 kDa respectively [19]. However, Ohashi *et. al*. showed that neither NRBF2 nor its yeast homolog Atg38 dimerized PtdIns3K-C1 based on their own SEC study [11]. Additionally, in a follow up study, Young *et. al*. showed that PtdIns3K-C1 requires the binding of NRBF2 dimer to be fully activated but only one binding site was observed in cryo-EM analysis [20]. Overall, *in vitro* binding studies and cryo-EM analysis favor the model of PtdIns3K-C1 containing two NRBF2 binding sites. Our co-IP data supports this model as the engineered dimeric or tetrameric NRBF2 constructs showed incrementally strengthened binding to PIK3R4/VPS15, probably through multivalent interactions engaging the two binding sites on PtdIns3K-C1. For the model of NRBF2-mediated dimerization of PtdIns3K-C1, our imaging data showed that engineered dimeric and tetrameric NRBF2 constructs formed distinct puncta *in vivo*, which superimposed with the autophagosome marker LC3. These findings suggest that NRBF2-mediated higher-order oligomerization of PtdIns3K-C1 is possible, especially under the context of early-stage autophagosome biogenesis that involves multimerization of autophagy markers like LC3. Further studies are needed to assess this model, especially under the *in vivo* setting.

NRBF2 exerts its proautophagic role by enhancing the lipid kinase activity of PtdIns3K-C1 [11–13]. How this effect is facilitated by the NRBF2-PIK3R4/VPS15 interaction is not fully understood. On the one hand, co-IP studies from several labs reveal that NRBF2 enhances the structural integrity of PtdIns3K-C1 as knockout of *Nrbf2* caused a fraction of PtdIns3K-C1 to dissociate into PIK3C3/VPS34-PIK3R4/VPS15 and BECN1-ATG14 subcomplexes [12,13]. Similar findings were also reported for the yeast homolog Atg38 [18]. On the other hand, cryo-EM studies suggest that NRBF2 allosterically modulates the structural flexibility of PtdIns3K-C1 and releases the catalytic domain of PIK3C3/VPS34 from its auto-inhibitory conformations to become fully active [20]. We propose that the oligomeric state of NRBF2 as mediated by its CC domain is critical for strengthening the moderate NRBF2-PIK3R4/VPS15 interaction for both scenarios so as to maintain the structural integrity of PtdIns3K-C1 or to allosterically activate PIK3C3/VPS34 within PtdIns3K-C1. With the cooperation of its MIT domain and CC domain, NRBF2 can fully activate PtdIns3K-C1 and promote autophagy.

## Materials and methods

### Reagents

Anti-ACTB/β-actin antibody (Santa Cruz Biotechnology, sc-47778), anti-Flag antibody (Cell Signaling Technology, 14793), anti-Flag M2 Magnetic Beads (Sigma-Aldrich, M8823), anti-GFP antibody (Cell Signaling Technology, 2555), anti-PIK3C3/VPS34 antibody (Cell Signaling Technology, 4263), anti-PIK3R4/VPS15 antibody (Bethyl Laboratories, A302-571A), anti-LC3 antibody (Novus, NB100–2220), anti-SQSTM1/p62 antibody (Abnova, H00008878-M01), Anti-Mouse IgG-HRP (Sigma-Aldrich, A9044), Anti-Rabbit IgG-HRP (Sigma-Aldrich, A9169), Lipofectamine 3000 (Thermo Fisher Scientific, L3000015), ELISA Class III PI3-Kinase Kit (Echelon Biosciences, K-3000), protease inhibitor cocktail (Roche Diagnostics, 4693132001), trypsin (Gibco, 15400054), isopropyl-β-D-thiogalactopyranoside (IPTG; Sigma-Aldrich, I6758), polyethylenimine (PEI; Yeasen, 40816ES02), 3-([3-cholamidopropyl] dimethylammonio)-1-propanesulfonate (CHAPS; Sigma-Aldrich, 1116620010), Bis-Tris buffer (Affymetrix, AFAJ1211222EA), Nonidet *p*-40 (Thermo Fisher Scientific, J19628AP), Triton X-100 (Sigma-Aldrich, 648466), biotin (IBA, 2–1016-005), ATP (Thermo Fisher Scientific, R0441), Strep-Tactin®XT resins (IBA, 2–5030-010), Ni^2+^-nitrilotriacetic acid (NTA) agarose beads (Invitrogen, 25215), PVDF membrane (Millipore, SEQ00010).

### Protein expression and purification

All NRBF2 constructs were generated by PCR and subcloned into a modified pET-32a vector (Novagen, 69015) containing the human rhinovirus 3C protease cleavage site and SUMO-His_6_ fusion. All constructs were expressed in *Escherichia coli* BL21 (DE3) cells at 30°C following induction with IPTG and purified by affinity chromatography (HisTrap HP; GE Healthcare, 17524802). The fusion tag was removed by HRV 3C protease (Sangon Biotech, C510303) cleavage and the untagged proteins were further purified by size-exclusion chromatography (Superdex 75; GE Healthcare, 28989333). The ATG14 and BECN1 fragments used for NMR titration and ITC experiments were expressed and purified following similar procedures. For the purification of full-length ATG14, Expi293F suspension cells (Thermo Fisher Scientific, A14527) were grown to around 3.0 × 10^6^ cells ml^−1^, and FLAG-Strep-ATG14 plasmids at 1.0 mg/L culture were transfected into cells using PEI, at a ratio of 3:1 PEI:DNA. Cells were harvested 3 days after transfection and resuspended in Buffer A (50 mM HEPES, pH 7.5, 200 mM NaCl, 5% glycerol, protease inhibitors) with 0.1% CHAPS and 1% Triton X-100. The cells were incubated at 4°C for 45 min on a shaker to fully extract the protein. Lysates were centrifuged at 21,000 × g and incubated with Strep-Tactin®XT resins for 4 h at 4°C. The resins were washed 4 times with Buffer A, 5 times with Buffer A with 800 mM NaCl and an additional 4 times with Buffer A. Proteins were eluted with buffer containing 100 mM Tris-HCl, pH 8.0, 150 mM NaCl, 1 mM EDTA and 50 mM biotin. The expression and purification of full-length BECN1, PIK3C3/VPS34 and PIK3C3/VPS34-PIK3R4/VPS15 subcomplex were expressed and purified following similar procedures.

### Affinity-isolation assay

SUMO-tagged NRBF2 protein was incubated with Ni-NTA agarose beads in binding buffer (50 mM Tris/HCl, pH 7.4, 150 mM NaCl) for 1 h at 4°C with rotation. SUMO tag was used as a control protein. The beads were washed 4 times with binding buffer to remove unbound proteins. Subsequently, the full-length ATG14, BECN1, PIK3C3/VPS34 and PIK3C3/VPS34-PIK3R4/VPS15 subcomplex were added respectively into the beads and incubated for 3 h at 4°C with rotation. After thorough washing with binding buffer, the bound proteins were eluted in SDS sample buffer and analyzed by SDS gel.

### Crystallization and structure determination

Crystals were grown at 16°C by the hanging drop vapor diffusion method mixing 1 μl of protein for NRBF2 CC domain at ~50 mg/ml with 1 μl of reservoir solution containing 3.92 M NaCl and 0.1 M Tris buffer (pH 7.0). The SeMet derivatives were obtained by mixing protein for NRBF2 CC Q171M mutant with reservoir solution containing 3.8 M NaCl and 0.1 M Bis-Tris buffer (pH 5.5). Data for native crystals were collected on the in-house X-ray system (MicroMax 007 HF, Rigaku), and data for SeMet derivatives were collected at beamline BL17U1 at the Shanghai Synchrotron Radiation Facility in Shanghai, People’s Republic of China. Statistics were summarized in Table S1. The coordinates of NRBF2 CC domain have been deposited to Protein Data Bank (PDB ID 9WT3).

### Isothermal titration calorimetry (ITC)

ITC assays were performed using an iTC200 microcalorimeter (MicroCal). All protein samples were dialyzed into 50 mM Tris, 150 mM NaCl at pH 8.0. For NRBF2-ATG14 and NRBF2-BECN1 interactions, the injection syringe was loaded with 40 μl of ATG14 or BECN1 sample and the cell was loaded with NRBF2 sample. Typically, titrations consisted of 16 injections with 180-s equilibration between injections. The data were analyzed using Origin 7.0.

### Circular dichroism (CD) spectroscopy

CD full-length scanning spectra were collected between 190 and 250 nm at room temperature using a Jasco J-810 spectropolarimeter equipped with thermoelectric temperature control. Protein samples were diluted to 50 μM in 50 mM NaCl, 20 mM Tris buffer, pH 8.0, and were loaded in a quartz cell with a 0.1-cm path length. Spectra were collected at 1.0 nm intervals with a 5-s averaging time per data point. A reference spectrum was collected from a scan of the buffer without protein. Melting temperature spectra were collected between 12 and 90°C at 222 nm. The temperature increment was 1°C, the equilibration time 3 min and the average time 1 min.

### Size-exclusion chromatography-light scattering

The size-exclusion chromatography-light scattering-UV (SEC-LS-UV) system was employed to detect the oligomeric state of NRBF2 proteins. 100 μg protein sample was applied to a Superdex 200 10/300 GL column (GE Healthcare, 17–5175-01). The injected volume was about 500 μl and the flow rate was 0.5 ml/min. The UV absorbance was detected at 280 nm while the light scattering signal was measured at 662 nm, and the final data were analyzed by software ASTRA (Wyatt technology).

### NMR titration studies

All ^15^N HSQC NMR experiments were performed at 298 K. Spectra were processed and analyzed using a Varian Inova 800 MHz spectrometer. ^15^N HSQC experiments were acquired with samples in 50 mM Tris, pH 7.4, 150 mM NaCl with 90% H_2_O:10% D_2_O. Concentration of ^15^N-labeled ATG14-1–95 was 0.1 mM. NMR titrations were performed by adding unlabeled concentrated NRBF2-MIT (0.1–0.8 mM) to ^15^N-labeled ATG14-1–95 (0.1 mM) gradually. The data were processed using NMRPipe and analyzed using Sparky.

### Cell culture

HEK293T (ATCC, CRL-3216) cells and HeLa cells with stable expression of RFP-GFP-LC3 or GFP-LC3 (gifts from Dr. Lu Jiahong, University of Macau, Taipa, Macau SAR, China) were cultured in Dulbecco’s modified Eagle’s medium (DMEM; Gibco, 11995073) supplemented with 10% fetal bovine serum (FBS; Gibco, 16000036). The WT and *nrbf2* KO N2a cells (gifts from Dr. Lu Jiahong) were cultured in DMEM:Opti-MEM (Gibco, 31985070; v:v = 1:1), 5% (v:v) FBS. Expi293F cells were cultured in SMM 293-TII Expression Medium (Sino Biological, M293TII). All cell lines used in the experiments were mycoplasma detected negative by MycoAlertTM PLUS Mycoplasma Detection Kit (Lonza, LT07-710) before and during the experiment.

### Immunoblot analysis

DNA plasmids were transiently transfected into cells using Lipofectamine 3000. For co-IP experiments, cells were lysed in IP buffer (25 mM HEPES PH 7.5, 10 mM MgCl_2_, 150 mM NaCl, 1 mM EDTA.2Na, 1% Nonidet *p*-40, 1% Triton X-100, 2% glycerol) with EDTA-free protease inhibitor cocktail. Protein lysates were incubated with anti-FLAG M2 magnetic beads overnight at 4°C. The beads were washed with 1× IP lysis buffer 5 times and then eluted with 2× SDS sample buffer. The eluted sample was subject to immunoblotting assay. For immunoblotting assay of SQSTM1 and LC3 degradation, GFP-NRBF2 mutant plasmids were transfected into *nrbf2* KO N2a cells for 24–48 h. Cells were lysed in Laemmli sample buffer (62.5 mM Tris-HCl, pH 6.8, 2% SDS, 25% glycerol, 5% β-mercaptoethanol) with EDTA-free protease inhibitor cocktail and cell lysate was directly subject to immunoblot assay.

### In vitro PIK3C3/VPS34 lipid kinase assay

*nrbf2* KO N2a cells transfected with FLAG-ATG14 and NRBF2 constructs were lysed in IP buffer (25 mM HEPES, pH 7.5, 10 mM MgCl_2_, 150 mM NaCl, 1 mM EDTA.2Na, 1% Nonidet *p*-40, 1% Triton X-100, 2% glycerol) with EDTA-free protease inhibitor cocktail. Immunoprecipitation was performed with Flag M2 beads. Immune complexes were washed 3 times in lysis buffer, followed by 2 times in Tris buffer (50 mM Tris-HCl, pH 7.5, 150 mM NaCl, 1 mM EDTA). A fraction (1/5) of the beads was aliquoted for western blot. Residual beads were resuspended in PIK3C3/VPS34 kinase reaction buffer (20 mM Tris, pH 8, 200 mM NaCl, 2 mM EDTA, 20 mM MnCl_2_, 100 μM ATP) and then incubated with PI substrate (Echelon Biosciences, K-3000) at 37°C for 60 min. PtdIns3P production was detected using an ELISA Class III PI3-Kinase Kit (Echelon Biosciences, K-3000).

### Fluorescence microscopy imaging

For the autophagic flux assay, HeLa cells stably expressing RFP-GFP-LC3 were transfected with FLAG-NRBF2 constructs. For the colocalization of NRBF2 and LC3, HeLa cell stably expressing GFP-LC3 were transfected with mCherry-NRBF2 constructs. After 24–48 h expression, these cells were processed for imaging study. Images were taken with 63x oil immersion objective lens of a confocal microscope and image acquisition was performed by LAS X software.

### Statistical analysis

For all quantitative data, mean ± SEM were shown, and *p* values < 0.05 were considered significant. To analyze the differences between two groups, unpaired t-test was used. For multiple group comparison, One-way analysis of variance (ANOVA) was used to test for statistical significance. Experiments were repeated at least 3 times for quantification.

## Supplementary Material

PDB_ValidationReport_18Sept2025.pdf

Supplemental_Figure_12Oct2025_Clean_R3v2.docx

## Data Availability

Data that support the findings of this study are included in this published article and its supplementary information files. Materials on this study are available from the corresponding author on reasonable request and might require a material transfer agreement.
