# Supplementary material for: NRBF2 homodimerization by its coiled-coil domain strengthens association with the PtdIns3K complex mediated by the MIT domain to promote autophagy
Source: Autophagy. 2025 Nov 12;22(1):168–81. doi: 10.1080/15548627.2025.2580438 (PMC12758336; doi:10.1080/15548627.2025.2580438)
Supplement: Supplemental_Figure_12Oct2025_Clean_R3v2.docx [file KAUP_A_2580438_SM1095.docx]

**Table S1.** Crystallographic data processing and refinement statistics.

^ Numbers in parenthesis define the highest resolution shell of data.

* Numbers in parenthesis are the statistics for the highest resolution shell of data.

**Figure S1.** Biochemical studies to assess whether NRBF2 binds to BECN1 or ATG14. (**A**) Constructs of ATG14 and BECN1 used in the binding assays. (**B and C**) Isothermal titration calorimetry (ITC) profiles to assess whether NRBF2 binds to ATG14 (B) or BECN1 (C). No bindings were detectable.

**Figure S2.** Biochemical studies to assess the oligomeric state of NRBF2 coiled coil domain. (**A**) Fast protein liquid chromatography (FPLC) coupled with static light scattering analysis measuring the oligomeric state of NRBF2 CC domain. The green plot is the elution profile. The fitted molecular mass of the fractions matching the NRBF2 CC domain is indicated by solid dots at the peak. (**B**) Temperature-dependent circular dichroism (CD) profile of NRBF2 CC domain at 220 nm. (**C**) The CD spectra of the NRBF2 CC domain at different temperatures. (B) and (C) suggest that the dimeric state of NRBF2 CC domain is highly stable.

**Figure S3.** Crystal structure of the NRBF2 CC domain. (**A**) The asymmetric unit of the crystal lattice contains eight copies of the helix representing the NRBF2 CC domain. (**B**) Snapshot of the 2Fo-Fc electron density map, contoured at 2σ level, that covers the NRBF2 1 CC domain. The dimeric protein structure is shown in Cα trace in green and yellow color.

**Figure S4.** Design of dimeric and tetramer NRBF2 CC domain using the Gcn4 motif. (**A and B**) Helical wheel presentation of the dimeric Gcn4-P1 (A) and the tetrameric Gcn4-P-L1 (B). Both Gcn4 constructs contain 5 heptad repeats and have the same sequence except those at *a* and *d* positions as highlighted.

**Figure S5.** Representative confocal fluorescence images of HeLa cells stably expressing GFP-LC3 after transfection with different NRBF2 constructs. GFP-LC3 puncta showed little overlap with monomeric NRBF2 constructs (3A and 5A) but overlapped significantly with NRBF2_Gcn4_Dimer and _Tetramer (scale bar: 10 μm).
